# Social Anxiety Disorder: Associations with Peer-Liking, Discrimination, and Prejudicial Feelings in Early Adolescent Girls

**DOI:** 10.1007/s10578-022-01336-5

**Published:** 2022-02-23

**Authors:** Ruth Brookman, Fay Bird, Celia B. Harris, Kerry-Ann Grant

**Affiliations:** 1 The MARCS Institute for Brain, Behaviour and Development, Western Sydney University, Locked Bag 1797, Penrith, NSW 2751 Australia; 2 KYDS Youth Development Service, 265 Pacific Hwy, Lindfield, NSW 2070 Australia; 3 Health Education and Training Institute, Locked Bag 7118, Parramatta BC, NSW 2124 Australia

**Keywords:** Social anxiety disorder, Early adolescence, Peer-liking, Stigma, Discrimination, Prejudicial feelings

## Abstract

Social anxiety can have an adverse effect on social connections, educational achievement, and wellbeing. However, the extent to which students stigmatize their peers with social anxiety disorder (SAD) in female educational settings remains unknown. This study investigated the relationship between SAD, peer-liking and stigma in a cohort of early adolescent girls. The sample was 103 sixth and seventh graders attending three girls’ schools in Australia. The students, aged between 10- and 13-years, were randomly allocated to either a control (*n* = 52) or experimental (*n* = 51) group. Participants completed an online survey while at school to examine their responses to one of two age-and-gender matched vignettes: a hypothetical peer with SAD (experimental condition), or without SAD (control condition). Contrary to expectations, group comparisons revealed that students with the SAD vignette liked their peer more than students with the non-SAD vignette. Also, students endorsed higher levels of pity, lower levels of fear, but similar levels of anger when considering their SAD (versus non-SAD) peer. In the SAD group, higher levels of pity were associated with greater peer-liking. The opposite pattern was evident in response to the non-SAD peer. Importantly, students discriminated less (preferred less social distance) in response to their peer with SAD. This points to the potential benefit of adolescent peer programs that aim to promote positive peer-relationships as a protective factor for students with SAD. Future research may examine gender and socio-economically diverse students to increase the confidence with which findings can be generalized to other educational settings.

## Introduction

Mental health difficulties in adolescents are prevalent and have reportedly risen in recent years with the prevalence of anxiety disorders surpassing that of depression [1, 2]. The onset of social anxiety, usually during adolescence, has been linked with adverse developmental outcomes in social, academic, physical and mental health domains [3]. As a broad construct, social anxiety exists on a continuum from subclinical feelings of nervousness to a clinical diagnosis of a mental health disorder [4]. According to the Statistical Manual of Mental Disorders, 5th edition (DSM-5) [5], a clinical diagnosis of social anxiety disorder (SAD), requires symptoms that cause significant distress and/or impairment to the individual affected [6]. The core symptom of SAD is a fear of being negatively evaluated by peers, that is often combined with difficulty functioning in social settings [7]. As such, the association between SAD and peer relationships has been examined in clinical [8], developmental [9], and social-cognitive psychological domains [10].

Social anxiety is most prevalent in western societies, females, and adolescents, with almost 1 in 10 adolescents affected [11]. Age-of-onset data specifically identifies *early adolesence* is a developmentally sensitive time period for the onset of SAD [12]. Early adolescence has been associated with greater abstract thinking and perspective taking [9], which could contribute to greater self-consciousness, fear of negative evaluation, and social avoidance [8]. In addition, prevalence data suggest social anxiety is most common amongst young females and young cohorts [1]. If left untreated, anxiety disorders can persist with an increased risk of educational underachievement, substance abuse disorders and anxiety disorders in adulthood. Further, SAD treatment has low efficacy rates in comparison to other anxiety disorders (e.g., [13]). This is the case regardless of treatment variations such as the inclusion of parents, homework completion, and group versus individual treatment (e.g., [14, 15]). However, it has also been suggested that adolescence is a developmental period of heightened flexibility and learning [1]. This raises a question as to whether early adolescence might be an appropriate time-period to target risks associated with SAD, especially in educational settings [1]. For these reasons, the present study is focused on the links between SAD, peer-stigma and peer-liking amongst early adolescent girls (10–13 years). To examine the possible role of peer relationships in contributing to the negative impacts of social anxiety, we used vignettes to examine how peers respond to their peers who exhibit socially-anxious behaviours. It is anticipated that findings will give direction to appropriate time periods for targeting interventions, particularly in female educational settings.

### SAD and Peer-Liking

It has been proposed that a number of factors can contribute to the etiology and maintenance of SAD, including biological, social and interpersonal influences (see [8] for a review). One example of a social influence is the role of peer relationships (see [8, 16, 17] for theoretical models). If socially anxious adolescents are *not* liked by their peers, there may be fewer opportunities for them to social engage with their peers. This together with social avoidance could contribute to the maintenance of anxiety symptoms for early adolescents. While there is consensus that adolescents with SAD perceive themselves as less likeable [18], there are mixed findings concerning their peer-rated likeability. Some studies suggest that children with anxiety disorders are *less* liked and experience more rejection than their non-anxious peers. Verduin and Kendall [19], for example, found that children with SAD (but not other anxiety disorders) were liked less by their peers after delivering a speech. Similarly, Barrow et al. [20] found that the children with SAD received lower peer-ratings, compared to the children without SAD. Baker et al. [21] conducted a similar study with 7–12-year-old actors who were instructed to give a verbal speech in either an anxious manner or in a non-anxious manner. The children who delivered the non-anxious (versus anxious) presentations received higher peer-rated liking scores. However, findings linking peer-liking and child social anxiety are mixed [22]. Baartmans et al. [18], for example, conducted a study with a large sample of 7–13-year-olds to find that children with higher levels of social anxiety were *more* liked by their peers than children with lower levels of social anxiety. These mixed results provide a challenge to drawing strong conclusions and raises a question about whether there is a moderating role of other variables such as peer-stigma.

### Stigmatization

Goffman [23] described stigma as a sign or a mark designating the bearer as flawed or compromised when compared to the non-stigmatized person. A common assumption in stigma theory is that individuals are categorized according to undesirable social stereotypes [24] that evoke prejudice [25]. For example, Weiner's [26] model proposes that stereotypes about personal responsibility for deviance can result in anger (when the individual is deemed responsible) or pity (when deemed blameless). Weiner's model was developed for adults but has been tested and validated in adolescents with respect to schizophrenia-related stigma [27] Although theoretical models of stigma differ, the construct is generally framed as a function of human cognition and discrimination (e.g., social rejection [28]). Corrigan [29] developed the attribution model of mental illness stigma, which is commonly applied in stigma research. The model predicts that appraising a person's behaviour as dangerous leads to fear, which in turn results in social avoidance and segregation. Social-cognitive attribution theories of stigma [26, 29] suggest that if we can identify the stereotypes, attributions and prejudicial feelings that children attach to peers with SAD, it may be possible to design interventions to challenge these assumptions and mitigate the risks of negative peer interactions [30].

Stigma theory also implicates the prejudicial feelings of pity, anger, and fear as causal mechanisms in the relationship between mental health problems and peer discrimination [26, 29]. Research examining stigmatization has therefore traditionally employed measures of prejudicial feelings (pity, anger, and fear) in addition to measures of discrimination (preference for social distance). While there is evidence that the prejudicial feelings of fear and anger are associated with depression [31], there is also evidence that this is not the case with feelings of pity [32, 33]. Overall, however, the *affective* components of peer-stigma and mental health disorders has received little research attention [34, 35]. Further, adolescent stigma studies are scarce and have largely focused on the stigmatization of adolescents with ADHD and depression [36]. A qualitative study by O’Driscoll et al. [31], for example, asked adolescents to imagine making friends with a peer diagnosed with ADHD. Participants anticipated that the peers’ impulsive behaviour would attract negative social consequences, leading to prejudicial feelings including anger, frustration, and embarrassment. Similarly, when considering a male hypothetical peer with depression, adolescents reported feelings of anger [32]. Further, Jorm and Wright [37] used one of four vignettes to examine stigma in youth (15–25 years) in response to different mental health conditions; depression, depression with alcohol misuse, social phobia, and schizophrenia. Results suggest that youth perceive their peers with social phobia (versus depression) to be weaker, but they do not discriminate against them more (i.e. prefer social distance). However, despite SAD being one of the most common mental health disorders in adolescence [6], the association between peer-stigma and SAD is unknown. This is surprising given the risk that peer-stigma can negatively impact psychological development [38].

Theoretical models of stigma together with empirical research examining SAD and peer stigma could inform the design of interventions that prevent stigmatizing attitudes and promote positive peer interactions in educational settings [39]. If negative peer responses are related to social anxiety in early adolescence and contribute to the aetiology and maintenance of SAD, then it is important to understand the mechanisms underlying these responses. Also, there may be unique ways in which stigma is expressed in all-girl educational settings. Jorm and Wright [37] for example, found that females endorsed lower levels of stigmatizing attributions and preference for social distance compared to males. Females have also been observed to express more benevolence and less discrimination and stigmatization toward those with mental health concerns (e.g., [40]). In contrast, males have been observed to blame peers for their depression when compared to their female counterparts [31]. This finding, together with the higher incidence of SAD in females, points to the value of examining the relationship between SAD in peer-stigma in female educational settings. Findings may assist with the development of gender specific interventions for this cohort and improve the management of SAD in female schools.

### The Present Study

Although evidence is mixed, the majority of the research evidence suggests that early adolescents with social anxiety are less liked by their peers [20]. However, to the authors’ knowledge, no studies have examined peer-stigma (discrimination and prejudicial feelings) in early adolescent girls. This is despite the high prevalence of SAD for this cohort [8], and the gender difference in discrimination in response to other mental health conditions [40]. The deleterious effect peer-stigma could have on recovery and help-seeking of early adolescent girls in female girl schools is unknown. The aim of this study was to examine the effect of SAD on peer-stigma and peer-liking in an educational setting of female early adolescents. To achieve this aim, all participants were randomly allocated to a SAD or non-SAD condition and asked to complete an online survey examining their responses to an age-and-gender matched vignette. Peer-stigma was examined using measures of discrimination (preference for social distance) and prejudicial feelings (pity, anger, and fear). To achieve our aim, we employed two complementary analytical approaches. First, we adopted a categorical approach to assess group differences (SAD versus non-SAD) in discrimination, and the affective components of peer-stigma: pity, anger, and fear. Second, we adopted a continuous approach to assess the relationship—in the SAD and non-SAD groups—between discrimination, peer-liking, and the prejudicial feelings of pity, anger and fear. This second analytical approach was exploratory.

Two primary predictions are made based on the literature reviewed.

1. SAD and peer-liking: compared to participants in the control group, early adolescent girls in the SAD group will endorse significantly lower levels of *liking* in response to a hypothetical peer vignette [19, 20].
2. SAD and peer-stigma: in response to a hypothetical peer vignette, early adolescent girls in the SAD (versus control) group will endorse significantly higher levels of the discrimination [31], fear and anger [31], but not pity [32, 33].

## Methods

### Participants

Participants were recruited from a convenience sample of private school girls by contacting parents of early adolescents attending one of three female educational settings—two secondary and one primary elementary school—located in Sydney, Australia. All families expressing interest were eligible to participate. One hundred and three early adolescent girls participated in this study. At the time of recruitment, participants’ age ranged from 10 to 13 years (Mean_age_ 11.96; SD_age_ 0.60). All participants attended a private school. Mean fees for grades 6 and 7, at the time of the study, were $20,592 per annum which was equivalent to approximately one-quarter of the average total annual cash earnings for full-time employees in Australia at the time (Australian Bureau of Statistics [ABS], 2014). No additional demographic information was obtained due to school privacy regulations.

Participants were randomly allocated to either experimental (*n* = 52) or control conditions (*n* = 51). Independent samples *t*-test confirmed that there were no significant differences in student age between experimental and control groups, *t*(101) = 0.33, *p* = 0.743. To control for the known effect of peer-raters’ social anxiety on their responding [21], children’s thoughts of social threat were measured (see ‘Materials and Measures’ below for details of the Social Threat Subscale of the Children’s Automatic Thoughts Scale [41]. An additional independent samples *t*-test confirmed no significant group difference in participants’ anxiety (thoughts of social threat, *t*(101) = − 0.53, *p* = 0.597.

The study was approved by the Macquarie University Human Research Ethics Committee approval number: 5201500195. All caregivers provided written informed consent prior to their adolescent child participating in the study. This research did not receive any specific grant from funding agencies in the public, commercial, or not-for-profit sectors.

### Materials and Measures

#### Vignettes

The vignettes described an age-and-gender matched early adolescent who engaged in behaviours that were either (a) consistent with a SAD diagnosis (experimental condition), or (b) inconsistent with a SAD diagnosis (control condition). The vignette used in the *experimental* condition was a social phobia vignette originally developed by Jorm et al. [42] and adapted by clinical researchers [43]. In the present study, the vignette was modified to describe an early adolescent that would meet DSM-5 [5] criteria for SAD (see ‘Appendix’). This included social avoidance, nervousness during class talks, and avoidance of eating in social situations. Further modifications include; removal of the reference to answering the telephone (to ensure the vignette was contemporary) and removal of the word “vomit” (to comply with the school principal’s request). The final version of the vignette was validated and rated for accuracy by a group of clinical researchers (*n* = 4) with experience working with adolescents and social anxiety, who provided a score from 1 (very inaccurate) to 6 (very accurate). Accuracy ratings (*M* = 4.25, *SD* = 1.5), support the validity and accuracy of the SAD vignette for use in the present study.

The vignette used in the *control* condition described an early adolescent female demonstrating behaviours that were not consistent with a SAD diagnosis, such as socializing with friends and playing group sports. The description of the control peer was an adaptation of the non-clinical vignette developed by Martin et al. [44] (see ‘Appendix’). The control vignette was validated and rated for accuracy as described above. Accuracy ratings (*M* = 5, *SD* = 0) support the validity and accuracy of the SAD vignette for use in the present study. Based on group feedback, the final version included the phrase “sometimes has a problem with feeling annoyed if she doesn't get her own way” (as opposed to “sometimes has some problems with needing to have her own way”).

To reduce the risk of bias, the name ‘Sarah’ was used for both vignettes and the self-reported measures below. ‘Sarah’ was a name that was not shared by any of the participants.

#### Peer-Liking

To measure peer ‘liking’ in relation to their hypothetical peer, students completed the 4-item Peer Liking Scale (PLS: [19]). Items were modified to refer to Sarah. An example item includes: “How much do you like Sarah?”. Items were rated on a 5-point scale ranging from 0 (not at all/definitely not) to 4 (very much/definitely), with higher scores indicating greater liking. Reliability of the PLS is good (*α* = 0.91 and 0.89; see [19, 20] respectively) and Cronbach's alpha indicated good internal consistency (*α* = 0.82) in the present study.

#### Peer-Discrimination

Peer-discrimination was operationalized as self-reported preference for social distance in relation to a hypothetical peer and was measured using the five-item Social Distance Scale (SDS: [45]), using a version modified for adolescents [37]. An example item is: ‘Would you be happy to work on a school project with Sarah?’ Items were rated on a 4-point scale from 1 (yes, definitely) to 4 (definitely not), with higher scores indicating greater preference for social distance from Sarah. Previous findings have established good reliability (*α* = 0.86 and 0.87; see [37, 46] respectively) and construct validity [45]. Cronbach's alpha indicated good reliability in the present sample (*α* = 0.83).

#### Prejudicial Feelings

Students self-reported their feelings of anger, fear, and pity in relation to their hypothetical peer. Each feeling was measured using a single item from the Revised Attribution Questionnaire (r-AQ: [47]). The r-AQ is comprised of the single items that loaded most strongly onto each of the factors identified in the 28-item Attribution Questionnaire. The r-AQ is valid and subscales for pity, anger, and fear have adequate reliability (α = 0.74, 0.89, and 0.96 respectively [47]). Each item included a self-statement in relation to the hypothetical peer (e.g., “Sarah makes me angry”). Scoring details were followed as specified in the Short Form for Children Attribution Questionnaire (AQ-8-C) [48]

#### Thoughts of Social Threat

To control for the known effect of peer-raters’ social anxiety on their responding [21], students’ thoughts of social threat were measured using the 10-item social threat subscale of the Children’s Automatic Thoughts Scale (CATS) [41]. An example item includes: “Everyone is staring at me.” Participants rated the frequency of thoughts in the past week using a 5-point scale from 0 (not at all) to 4 (all the time), with higher scores indicating more automatic thoughts of social threat. The subscale has demonstrated good discriminant validity and internal consistency (α = 0.92), and adequate test–retest reliability at 3 months (*r* = 0.73) [41]. Reliability of the CATS in the current sample was good (*α* = 0.92).

### Pilot

The online survey was piloted in a small sample of age-matched early adolescent girls (n = 6). Pilot participants answered questions assessing their understanding of instructions and questions, and whether they found the questionnaire tiring to complete. Responses indicated that the wording and length of the survey were age and content appropriate.

### Procedure

Data collection took place on-location at two secondary and one primary elementary educational setting during class time. Prior to accessing the questionnaire, participants watched a short PowerPoint presentation of survey instructions, such as details of how to record their ratings. Participants then received an envelope containing a number and a randomly assigned unique identifier to allocate them to either the Experimental (SAD) or Control (non-SAD) condition. Participants used their own devices to access a school-issued email that contained a link to the online survey. Students were prompted to enter their age and identifier prior to completing the survey. Two information technology staff, a researcher and a teacher were present to provide instructions and assist participants with technical issues and questions.

## Results

### Descriptive Statistics

Descriptive statistics for all measures for the Experimental and Control conditions are displayed in Table 1. Preliminary *t*-tests assessed the effects of participant’s age on discrimination and prejudicial feelings measures, and the relations of these measures to student’s thoughts of social threat (control measure). As all results were non-significant ‘age’ and ‘thoughts of social threat’ were not included in subsequent analyses. All data were screened for outliers, and statistical assumptions were deemed satisfactory.

**Table 1:** Descriptive statistics for peer-reported liking, peer-discrimination, and prejudicial feelings across control (non-SAD) and experimental (SAD) groups

	Control (*n* = 52)		Experimental (*n* = 51)	
	*M*	*SD*	*M*	*SD*
Peer-liking	9.35	3.16	11.04	2.78
Discrimination	2.42	0.56	1.95	0.60
PF 1—pity	3.67	1.98	7.35	1.57
PF 1—anger	1.87	1.33	1.78	1.39
PF 2—fear	1.71	1.61	1.43	1.60

*Discrimination* preference for social distance; *PF* prejudicial feelings

### The Social Consequences of SAD

We examined whether there were between group differences in peer-liking, discrimination and prejudical feelings based on whether or not the hypothical peer ‘Sarah’ exhibited symptoms of social anxiety. This analysis indicated that students with the SAD vingnette differed in their response to their hyothetical peer with regard to the majority of affective stigma responses (liking, pity and fear) and also their preference for social distance from the peer (discrimination). An adjusted *t*-statistic is reported in cases where Leven’s test showed significant departure from equality of variances.

### Do Students in Female Schools Like Their Peers with Social Anxiety?

An independent samples *t*-test was conducted to test for group differences in peer liking. Contrary to our prediction, the significant group difference, *t*(101) = 2.89, *p* = 0.005 indicated that students in the SAD group did not endorse lower levels of liking in response to their vignette. Results indicated that students in the SAD group expressed higher levels of liking in response to their hypothetical peer (*M* = 11.04, *SE* = 0.39) compared to students in the control group (*M* = 9.35, *SE* = 0.44).The difference of 1.69, BCa 95% CI 0.44–3.04 represented a medium-sized effect, *d* = 0.57 [49].

In conclusion, students with the SAD vignette liked ‘Sarah’ more, compared to students with the non-SAD vignette.

### Do Students in Female Schools Stigmatise Their Peers with Social Anxiety?

#### Peer-Discrimination

Next, to test the prediction that children in the SAD (versus non-SAD) group would endorse more discrimination in response to their hypothetical peer, an independent samples *t*-test was conducted. Contrary to our prediction the significant group difference, *t*(101) = − 4.12, *p* < 0.001, indicated that students in the control group (*M* = 2.42, *SE* = 0.08) demonstrated higher levels of discrimination toward their non-SAD vignette (*M* = 1.95, *SE* = 0.08) compared to students in the experimental group toward their SAD vignette. This difference of 0.47, BCa 95% CI − 0.68, − 0.27, represented a large-sized effect, *d* = 0.81 [49].

In conclusion, students with the SAD vignette endorsed less discrimination (preference for social distance from ‘Sarah’) compared to students with the non-SAD vignette.

#### Prejudicial Feelings

Finally, to test the prediction that early adolescent girls in the SAD group would endorse significantly higher levels of anger and fear but not pity compared to controls, one independent samples *t*-test and two Mann–Whitney *U* tests were conducted (see Table 2). Unexpectedly, there was a significant group difference in pity, *t*(96.89) = 10.46, *p* < 0.001, with higher levels in the SAD group (*M* = 7.35, *SE* = 0.22), compared to the control group (*M* = 3.67, *SE* = 0.27). This difference, 3.68, BCa 95% CI 2.96–4.41 exceeded two standard deviations, representing a large effect size, *D* = 2.06 [49]. Contrary to the prediction, there was no significant group difference comparing anger, *U* = 1395.50, *p* = 0.598, *r* = 0.05, in the Control (mean rank = 53.34) and SAD (mean rank = 50.64) groups. There was a significant group difference in levels of *fear*, *U* = 1544.50, *p* = 0.040, *r* = 0.20, in the control group (mean rank = 56.20), and SAD group (mean rank = 47.72). In contrast to our prediction, however, the direction of the difference indicated that fear was highest in the control group.

**Table 2:** Inferential statistics for peer-liking, discrimination, and prejudicial feelings across control (non-SAD) and experimental (SAD) groups

Variable—mean (SD)	Group		*t*	*df*	*p*
	Control (*n* = 52)	Experimental *(n* = 51)			
Peer-liking	9.35 (3.16)	11.04 (2.78)	2.89	101	0.005**
Discrimination	2.42 (0.56)	1.95 (0.60)	− *4.12*	101	< 0.001***
PF 1—pity	3.67 (1.98)	7.35 (1.57)		96.89	< 0.001***

Variable—mean rank			*U*	*Sig.*
PF 2—anger	53.34	50	1395.50	0.598
PF 3—fear	56.20	47.72	1544.50	0.04*

*Discrimination* preference for social distance; *PF* prejudicial feelings

*p < 0.05; **p < 0.01; ***p < 0.001

In summary, students with the SAD vignette endorsed greater feelings of pity, similar levels of anger, and lower levels of fear compared to students with the non-SAD vignette.

### Is There a Relationship Between Discrimination, Liking and Prejudical Feelings?

Pearson correlations were carried out to determine whether discrimination was related to peer-liking and prejudical feelings, for students in the experimental (SAD) and control (non-SAD). In both groups, peer-liking was negatively and significantly negatively correlated with students’ discrimination. This finding indicates that as peer-liking increased, discrimination decreased, regardless of whether students were given the SAD, *r*(51) = − 0.739, *p* = 0.000, or the non-SAD vignette, *r*(52) = − 0.654, *p* = 0.000.

Discrimination and peer-liking were not significantly correlated with the affective stigma responses of anger and fear. This was the case in both the SAD and non-SAD groups (see Table 3). However, there were some key group differences in the pattern of relationships between peer-liking, discrimination, and the affective stigma response of pity. There was an interesting divergence in the relationships between pity and peer-liking according to vignette type (SAD or non-SAD). In the experimental (SAD) group, pity was significantly associated with peer-liking, *r*(51) = 0.400, *p* = 0.004 whereby higher levels of pity were associated with greater peer-liking. In contrast, in the control group, higher levels of pity were associated with less peer-liking, albeit with a very weak negative relationship *r*(52) = − 0.091, *p* = 0.520. A similar divergent pattern was evident in the association between feelings of pity and discrimination. In the experimental (SAD) group, pity was negatively associated with discrimination, whereby higher levels of pity were associated with lower levels of discrimination in response to the SAD vignette, with a correlation score that approached significance *r*(51) = − 0.274, *p* = 0.051. In contrast, the control group demonstrated a positive association between pity and discrimination, whereby higher levels of pity were associated with more discrimination, again with a weak relationship *r*(52) = 0.112, *p* = 0.430.

**Table 3:** Results of correlation analyses (Pearson *r*) including prejudicial feelings, discrimination, and peer-liking across control (non-SAD) and experimental (SAD) groups

	Anger	Fear	Discrimination	Peer-liking
Control group (*n* = 52)				
PF 1—pity	0.140	0.124	0.112	− 0.091
PF 2—anger		0.100	0.256	− 0.185
PF 3—fear			0.254	− 0.026
Discrimination				− 0.654***
Experimental group (*n* = 51)				
PF 1—pity	− 0.193	− 0.193	− 0.274	0.400**
PF 2—anger		− 0.002	0.035	− 0.050
PF 3—fear			0.144	− 0.202
Discrimination				− 0.739***

*PF* prejudicial feelings; *Discrimination* preference for social distance

**p < 0.01; ***p < 0.001

## Discussion

There is mounting evidence to suggest that the prevalence of mental health concerns is rising as a result of the global COVID-19 pandemic [50, 51]. The social consequence of mental health disorders during early adolescence can impact negatively on disclosure and the help-seeking behaviour of students. This study is the first to examine whether early adolescent girls discriminate against age-and-gender matched peers with SAD by disliking them and avoiding social interaction with them. This study also examined whether young adolescent girls have unique affective stigma responses to peers with SAD that might be linked with discrimination and liking. In the SAD group higher levels of pity were associated with greater peer-liking. The opposite pattern was evident in response to the non-SAD peer. Although non-significant, a similar divergent pattern was evident in the association between feelings of pity and discrimination in the SAD group. Overall, our results suggest that early adolescent girls with SAD are more liked, more pitied, less feared, and less discriminated against by their peers (in terms of desire for social interaction) than those without SAD.

Our finding that early adolescent girls did not endorse higher levels of *discrimination* in response to their hypothetical SAD peer, was contrary to our prediction. However, when examined together with the unexpected affective stigma responses—high levels of pity and liking—this finding is congruent with theoretical frameworks of stigma. Weiner's theory [26], for example, suggests that sympathetic peer-responses to people with psychological problems evokes feelings of liking and acceptance, whereas angry responses will result in social distance and rejection. Juvonen [33] tested this theory by examining the relationship between perceived deviance, responsibility, liking, attribution-dependent emotions (pity, sympathy, irritation, and anger), social rejection, and social support in a sample of 12 year old Finnish children. Results suggested that children's perception of deviance was related to social rejection and that peer-rejection varied as a function of their attribution of responsibility i.e. feelings of anger (if the individual is deemed responsible for their behaviour) or pity (if the individual is deemed blameless). Similarly, Dolphin and Hennessy [32] found that when an individual with depression is perceived by peers as having little or no control over the cause of their condition, then responsibility is not inferred. Rather, they found that feelings of sympathy and pity were evoked, and social acceptance was more likely. It could be that the hypothetical peer described in the SAD vignette in the current study was deemed blameless for their anxious behaviours, thus evoking the higher levels of pity and liking and lower levels of discrimination observed.

Although findings have been mixed, the higher levels of peer-liking in the present study are consistent with several studies linking anxiety and *greater* peer-liking [18, 22, 52]. These mixed findings could be attributed to the lack of homogeneity in methodological approaches to measuring ‘peer-likeability’, such as using peer nominations of most-liked and least-liked classmates [18] or peer-liking ratings of children giving oral presentations in an anxious and non-anxious manner [21]. In the present study, adolescents were asked to consider a hypothetical vignette. A strength of this approach was that it enabled the experimental condition to be defined according to DSM-5 diagnostic criteria for SAD symptoms [5]. This was considered advantageous as diagnostic criteria are considered to be consistent and reliable yielding improved accuracy and reduced risk of misclassification such as those associated with high comorbidity in clinical samples [19]. However, unlike the methodology of using oral presentations, vignettes include contextual information that can potentially influence participants’ responses. For example, the SAD vignette used in the present study suggested motivation for Sarah’s anxious behaviour (i.e. ‘she is scared that she’ll do or say something embarrassing’). This contextual information could have evoked affective responses in raters such as greater liking and pity, and less fear. A higher endorsement of peer-liking may in turn influence lower levels of discrimination for some participants. This possibility is suggested by our finding that in the experimental (SAD) group, but not in the control group, higher levels of pity were associated with greater peer liking and with lower levels of discrimination.

The use of a hypothetical SAD vignette could also have influenced other affective peer responses in our study, such as lower levels of fear, and higher levels of pity. A study with adolescents and young adults by Yap et al. [46], found that accurate labels of mental health disorders were associated with less peer-stigma. While labels were not used explicitly in the present study, the SAD vignette’s description of Sarah’s avoidance behaviours together with the reason given for her avoidance, may have lowered stigma responses. Also, other studies have examined peer attractiveness with SAD [20] to find that physical attractiveness is a stronger predictor of peer-liking than anxiety. This raises the question of whether other factors, in addition to SAD, may have contributed to the positive affective peer-ratings observed in the present study.

It is possible that the gender of our sample and links with empathy might have contributed to the high levels of positive peer-responding observed. It has been observed that patterns of stigmatizing responses may vary according to the gender of respondents [31, 37]. Female respondents, for example, tend to express more benevolence and less discrimination and stigmatization toward those with mental health concerns (e.g., [37, 40]). In contrast, males tend to blame peers for their depression when compared to their female counterparts [31]. It could also be that early adolescent girls are particularly likely to empathize with their socially anxious peers, and that this empathy could motivate a desire for social connection. Higher levels of empathy in young adults is associated with lower incidences of using stigmatizing nouns to label others (e.g., [53]) and interventions aimed at increasing empathy, also see a reduction in stigma [54]. Our study with early adolescent girls, raises a question regarding the role of empathy in the relationship between SAD and peer-stigma. While the examination of empathy is beyond the scope of the present study, this variable would be a fruitful direction for future research in relationship to gender and mixed educational settings.

Rather than discrimination, peers in the present study endorsed a *preference* for social interaction with a peer with SAD. Interestingly, this pattern has not been evident in research examining other mental health conditions e.g., amongst peers with ADHD or depression [31]. Similarly, Jorm and Wright [37] found that psychosis, compared to depression, elicited greater peer-responses of preference for social distancing and Walker et al. [55] found that a diagnosis of depression was associated with greater stigma than one of ADHD. In contrast, O’Driscoll et al. [31] reported that a hypothetical peer with ADHD attracted greater peer-stigma responses than a peer with depression. However, these results were based on explicit (vs. implicit) measures of stigma. It is unknown whether this pattern of responding with SAD would be similar if implicit stigma measures were employed. Taken together, these results highlight the importance of examining peer-stigma in relation to a broad range of mental health conditions, including social anxiety.

The promotion of positive peer relationships in female educational settings may be an important factor for mitigating risk associated with social anxiety during early adolescence. The extant literature suggests that close friendships serve as a protective factor against the development and maintenance of social anxiety [56] and that strategies to support peer-friendships have a positive influence on social anxiety [57]. Studies have shown that general peer acceptance is associated with lower levels of internalizing problems during childhood and adolescence (e.g., [58]). Specifically, close friendships are associated with a decrease in symptomatology over time [59] and the quality of friendships predicts better treatment outcomes for SAD [60]. There is opportunity for future research to consider mixed methods approaches to further examine the relationship between peer-stigma and SAD, and the potential benefits of promoting positive peer relationships in female educational settings.

## Limitations and Future Research

There are methodological aspects to the study that could have influenced the findings reported here. The non-SAD vignette, for example, includes the statement “Sarah … sometimes has a problem with feeling annoyed if she doesn’t get her way” to provide detail regarding typical “non-clinical” problems for this peer. As this statement is not included in the SAD vignette, however, it may have contributed to the non-SAD being perceived as less likeable. Similarly, the SAD vignette includes detail concerning Sarah’s motivation for her anxious behaviour i.e., “She [Sarah] is scared that she’ll do or say something embarrassing when she’s around others.” The disclosure of Sarah’s internal motivation may have evoked empathy in participants and higher levels of pity and liking and lower preferences for social distance and fear. Future research could therefore explore the role of internal motivations in influencing peer-responses to SAD. For example, conducting a replication of the current study using vignettes that either contain or exclude internal motivations for socially anxious behaviours. Additional measures examining the validity of the vignettes could also address the above limitation. Validity information was sought through vignette accuracy ratings provided by a panel of experts for the SAD and non-SAD vignettes. A limitation of the presents study is that the accuracy ratings provided by the panel may not reflect true validity of the task. It is recommended, therefore, that future vignette-studies consider employing additional validation measures.

It is acknowledged that participants’ responses to the peer vignettes may not mirror their actual behavioural responses in real social situations. While self-report and attitudinal studies provide valuable information about stigma, it is in the context of social interactions that peer-stigmas are formed. Future research could therefore consider the examination of peer-stigma using real-life social situations which provide participants with additional social information such as their peer’s temperament, interests, attitudes and peer group [61]. The use of technology such as 3-D virtual reality could provide an opportunity for the SAD peer’s anxious behaviours to be displayed and/or interacted with by the peer-rater. Using such technology could enable responses to be manipulated within vignettes to account for real-life variables such as social consequences. Manipulating the hypothetical peer’s acceptance or approval of the peer-rater’s avoidant or approach behaviour toward the SAD peer could provide a more nuanced indicator of factors that influence peer-liking and/or stigma of individuals with SAD.

As a preliminary study, the sample size was sufficient to reveal significant effects and were suitable for the statistical analyses reported. A larger sample size, however, may have allowed us to conduct sub-group analyses comparing SAD with other anxiety disorders and to gain further insights into the associations between SAD, peer-liking and peer-stigma. Including a measure of stigmatizing attitudes towards mental illness more broadly within our sample would have enabled comparison with norms (where available) or results from other studies. Also, a threat to the generalization of these findings is the use of a convenience sample that included educational settings with full-time psychologists on-staff. Prosocial attitudes and mental health literacy in the school environment could have influenced the findings here. Future research could consider the use of diverse gender and socio-economic sample groups.

## Summary

Social anxiety can have an adverse effect on social connections, educational achievement, and wellbeing. However, the extent to which students stigmatize their peers with SAD in female educational settings was previously unknown. The present study extends the existing SAD literature by investigating the relationship between SAD, peer-liking and stigma in a cohort of 103 early adolescent girls aged between 10- and 13-years. Participants were randomly allocated into a control or experimental group where and completed an online survey in response to a control or SAD vignettes. Contrary to expectations, group comparisons revealed that students with the SAD vignette liked their peer more than controls and endorsed higher levels of pity, lower levels of fear, but similar levels of anger. In the SAD group, higher levels of pity were associated with greater peer-liking. The opposite pattern was evident in response to the non-SAD peer. Importantly, students discriminated less (preferred less social distance) in response to their peer with SAD. These findings have the potential inform the development of appropriate interventions including the promotion of positive peer relationships in female educational settings. Future research should consider the use of gender and socio-economically diverse samples economically diverse students to increase the confidence with which findings can be generalized to other educational settings.

## Appendix

### Vignette 1: Experiment (SAD) Condition

Sarah is in the same year as you. Kids at her school say that she is really shy. She often sits by herself during recess and lunch because she is scared that she’ll do or say something embarrassing when she’s around others. Also, she doesn’t like to eat in front of other people. Sarah has one close friend. Although Sarah does well at school, she hardly ever says a word in class and becomes incredibly nervous, trembles, blushes and looks like she is feeling ill if she has to answer a question or speak in front of the class. Sarah also finds it hard to talk if she has to meet anyone new. She refuses to join any clubs or group or go to parties or discos.

### Vignette 2: Control (non-SAD) Condition

Sarah is in the same year as you. She likes her school because she has good friends in her class but complains about having to do too much homework. She usually hangs out with her friends at the weekend. She has several hobbies, including playing sports and listening to music. Sarah usually gets on well with other kids, but sometimes has a problem with feeling annoyed if she doesn't get her own way. Sarah does well at school—although she has to work hard at subjects that she finds difficult. She is well-behaved in school and hardly ever gets in trouble from her teacher.

## References

[1] Lawrence D, Hafekost J, Johnson SE, Saw S, Buckingham WJ, Sawyer MG, Ainley J, Zubrick SR (2016). Key Findings from the second Australian child and adolescent survey of mental health and wellbeing. Aust N Z J Psychiatry.

[2] Wagner G, Zeiler M, Waldherr K, Philipp J, Truttmann S, Dür W, Treasure JL, Karwautz AF (2017). Mental health problems in Austrian adolescents: a nationwide, two-stage epidemiological study applying DSM-5 criteria. Eur Child Adolesc Psychiatry.

[3] Rapee RM, Schniering CA, Hudson JL (2009). Anxiety disorders during childhood and adolescence: origins and treatment. Annu Rev Clin Psychol.

[4] Beidel DC, Turner SM, Morris TL (1999). Psychopathology of childhood social phobia. J Am Acad Child Adolesc Psychiatry.

[5] Association AP (2013). Diagnostic and statistical manual of mental disorders (DSM-5®).

[6] Rao PA, Beidel DC, Turner SM, Ammerman RT, Crosby LE, Sallee FR (2007). Social anxiety disorder in childhood and adolescence: descriptive psychopathology. Behav Res Ther.

[7] Asbrand J, Heinrichs N, Schmidtendorf S, Nitschke K, Tuschen-Caffier B (2020). Experience versus report: where are changes seen after exposure-based cognitive-behavioral therapy? A randomized controlled group treatment of childhood social anxiety disorder. Child Psychiatry Hum Dev.

[8] Rapee RM, Spence SH (2004). The etiology of social phobia: empirical evidence and an initial model. Clin Psychol Rev.

[9] Cicchetti J, Cohen DJ (1995). Developmental psychopathology: theory and methods.

[10] Kingery JN, Erdley CA, Marshall KC, Whitaker KG, Reuter TR (2010). Peer experiences of anxious and socially withdrawn youth: an integrative review of the developmental and clinical literature. Clin Child Fam Psychol Rev.

[11] Burstein M, He JP, Kattan G, Albano AM, Avenevoli S, Merikangas KR (2011). Social phobia and subtypes in the national comorbidity survey-adolescent supplement: prevalence, correlates, and comorbidity. J Am Acad Child Adolesc Psychiatry.

[12] Haller SP, Kadosh KC, Scerif G, Lau JY (2015). Social anxiety disorder in adolescence: how developmental cognitive neuroscience findings may shape understanding and interventions for psychopathology. Dev Cogn Neurosci.

[13] Hudson JL, Rapee RM, Lyneham HJ, McLellan LF, Wuthrich VM, Schniering CA (2015). Comparing outcomes for children with different anxiety disorders following cognitive behavioural therapy. Behav Res Ther.

[14] Lundkvist-Houndoumadi I, Thastum M, Nielsen K (2016). Parents’ difficulties as co-therapists in CBT among non-responding youths with anxiety disorders: parent and therapist experiences. Clin Child Psychol Psychiatry.

[15] Arendt K, Thastum M, Hougaard E (2016). Homework adherence and cognitive behaviour treatment outcome for children and adolescents with anxiety disorders. Behav Cogn Psychother.

[16] Crick NR, Dodge KA (1994). A review and reformulation of social information-processing mechanisms in children’s social adjustment. Psychol Bull.

[17] Rubin KH, Burgess KB, Vasey MW, Dadds MR (2001). Social withdrawal. The developmental psychopathology of anxiety.

[18] Baartmans JMD, Rinck M, Hudson JL, Lansu TAM, van Niekerk RE, Bögels SM, Klein AM (2019). Are socially anxious children really less liked, or do they only think so?. Cogn Ther Res.

[19] Verduin TL, Kendall PC (2008). Peer perceptions and liking of children with anxiety disorders. J Abnorm Child Psychol.

[20] Barrow ME, Baker JR, Hudson JL (2011). Peer liking, physical attractiveness, and anxiety disorders in children. J Exp Psychopathol.

[21] Baker JR, Hudson JL, Taylor A (2014). An investigation into the lower peer liking of anxious than nonanxious children. J Anxiety Disord.

[22] Crick NR, Ladd GW (1993). Children’s perceptions of their peer experiences: attributions, loneliness, social anxiety, and social avoidance. Dev Psychol.

[23] Goffman E (1963). Stigma and social identity. Underst Deviance Connect Class Contemp Perspect.

[24] Hamilton DL, Stroessner SJ, Driscoll DM, Devine PG, Hamilton DL, Ostrom TM (1994). Social cognition and the study of stereotyping. Social cognition: impact on social psychology.

[25] Weiner B, Perry RP, Magnusson J (1988). An attributional analysis of reactions to stigmas. J Pers Soc Psychol.

[26] Weiner B, Martinko M, Martinko MJ (1995). Attribution theory in organizational behavior: a relationship of mutual benefit. Attribution theory: an organizational perspective.

[27] Corrigan PW, Watson AC (2007). How children stigmatize people with mental illness. Int J Soc Psychiatry.

[28] Link BG, Phelan JC (2001). Conceptualizing stigma. Annu Rev Sociol.

[29] Corrigan PW (2000). Mental health stigma as social attribution: implications for research methods and attitude change. Clin Psychol Sci Pract.

[30] Corrigan PW, Morris SB, Michaels PJ, Rafacz JD, Rüsch N (2012). Challenging the public stigma of mental illness: a meta-analysis of outcome studies. Psychiatr Serv.

[31] O’Driscoll C, Heary C, Hennessy E, McKeague L (2012). Explicit and implicit stigma towards peers with mental health problems in childhood and adolescence. J Child Psychol Psychiatry.

[32] Dolphin L, Hennessy E (2014). Adolescents’ perceptions of peers with depression: an attributional analysis. Psychiatry Res.

[33] Juvonen J (1991). Deviance, perceived responsibility, and negative peer reactions. Dev Psychol.

[34] Hinshaw SP (2005). The stigmatization of mental illness in children and parents: developmental issues, family concerns, and research needs. J Child Psychol Psychiatry.

[35] McKeague L, Hennessy E, O’Driscoll C, Heary C (2015). Retrospective accounts of self-stigma experienced by young people with attention-deficit/hyperactivity disorder (ADHD) or depression. Psychiatr Rehabil J.

[36] Mukolo A, Heflinger CA, Wallston KA (2010). The stigma of childhood mental disorders: a conceptual framework. J Am Acad Child Adolesc Psychiatry.

[37] Jorm AF, Wright A (2008). Influences on young people’s stigmatising attitudes towards peers with mental disorders: national survey of young Australians and their parents. Br J Psychiatry.

[38] Kaushik A, Kostaki E, Kyriakopoulos M (2016). The stigma of mental illness in children and adolescents: a systematic review. Psychiatry Res.

[39] Heary C, Hennessy E, Swords L, Corrigan P (2017). Stigma towards mental health problems during childhood and adolescence: theory, research and intervention approaches. J Child Fam Stud.

[40] Dey M, Paz Castro R, Jorm AF, Marti L, Schaub MP, Mackinnon A (2020). Stigmatizing attitudes of Swiss youth towards peers with mental disorders. PLoS ONE.

[41] Schniering CA, Rapee RM (2002). Development and validation of a measure of children’s automatic thoughts: the children’s automatic thoughts scale. Behav Res Ther.

[42] Jorm AF, Wright A, Morgan AJ (2007). Beliefs about appropriate first aid for young people with mental disorders: findings from an Australian National Survey of Youth and Parents. Early Interv Psychiatry.

[43] Lubman DI, Hides L, Jorm AF, Morgan AJ (2007). Health professionals’ recognition of co-occurring alcohol and depressive disorders in youth: a survey of Australian general practitioners, psychiatrists, psychologists and mental health nurses using case vignettes. Aust N Z J Psychiatry.

[44] Martin JK, Pescosolido BA, Olafsdottir S, McLeod JD (2007). The construction of fear: Americans’ preferences for social distance from children and adolescents with mental health problems. J Health Soc Behav.

[45] Link BG, Phelan JC, Bresnahan M, Stueve A, Pescosolido BA (1999). Public conceptions of mental illness: labels, causes, dangerousness, and social distance. Am J Public Health.

[46] Yap MB, Mackinnon A, Reavley N, Jorm AF (2014). The measurement properties of stigmatizing attitudes towards mental disorders: results from two community surveys. Int J Methods Psychiatr Res.

[47] Corrigan P, Markowitz FE, Watson A, Rowan D, Kubiak MA (2003). An attribution model of public discrimination towards persons with mental illness. J Health Soc Behav.

[48] Corrigan PW, Lurie BD, Goldman HH, Slopen N, Medasani K, Phelan S (2005). How adolescents perceive the stigma of mental illness and alcohol abuse. Psychiatr Serv.

[49] Cohen J (1988). Statistical power analysis for the behavioral sciences.

[50] Ravens-Sieberer U, Kaman A, Erhart M, Devine J, Schlack R, Otto C (2021). Impact of the COVID-19 pandemic on quality of life and mental health in children and adolescents in Germany. Eur Child Adolesc Psychiatry.

[51] Li SH, Beames JR, Newby JM, Maston K, Christensen H, Werner-Seidler A (2021). The impact of COVID-19 on the lives and mental health of Australian adolescents. Eur Child Adolesc Psychiatry.

[52] Bell-Dolan DJ (1995). Social cue interpretation of anxious children. J Clin Child Psychol.

[53] Howell AJ, Ulan JA, Powell RA (2014). Essentialist beliefs, stigmatizing attitudes, and low empathy predict greater endorsement of noun labels applied to people with mental disorders. Pers Individ Differ.

[54] Devine PG, Forscher PS, Austin AJ, Cox WT (2012). Long-term reduction in implicit race bias: a prejudice habit-breaking intervention. J Exp Soc Psychol.

[55] Walker JS, Coleman D, Lee J, Squire PN, Friesen BJ (2008). Children’s stigmatization of childhood depression and ADHD: magnitude and demographic variation in a national sample. J Am Acad Child Adolesc Psychiatry.

[56] La Greca AM, Harrison HM (2005). Adolescent peer relations, friendships, and romantic relationships: do they predict social anxiety and depression?. J Clin Child Adolesc Psychol.

[57] Erath SA, Flanagan KS, Bierman KL (2007). Social anxiety and peer relations in early adolescence: behavioral and cognitive factors. J Abnorm Child Psychol.

[58] Waldrip AM, Malcolm KT, Jensen-Campbell LA (2008). With a little help from your friends: the importance of high-quality friendships on early adolescent adjustment. Soc Dev.

[59] Van Zalk N, Van Zalk M (2015). The importance of perceived care and connectedness with friends and parents for adolescent social anxiety. J Pers.

[60] Baker JR, Hudson JL (2013). Friendship quality predicts treatment outcome in children with anxiety disorders. Behav Res Ther.

[61] Nangle DW, Erdley CA, Zeff KR, Stanchfield LL, Gold JA (2004). Opposites do not attract: social status and behavioral-style concordances and discordances among children and the peers who like or dislike them. J Abnorm Child Psychol.

